# Supplementary material for: A Transcriptomics-Based Bioinformatics Approach for Identification and In Vitro Screening of FDA-Approved Drugs for Repurposing against Dengue Virus-2
Source: Viruses. 2022 Sep 29;14(10):2150. doi: 10.3390/v14102150 (PMC9609047; doi:10.3390/v14102150)
Supplement: Supplementary file 1 [file viruses-14-02150-s001.zip › Supplementary tables (2).pdf]

Table S1 Signature genes identified using GEO datasets

Sr. No	Gene List
1	AAG5
2	ABCA5
3	ABCB9
4	ABCC13
5	ABLIM1
6	ACACB
7	ACAT1
8	ACBD5
9	ACOT7
10	ACOX1
11	ACTG1
12	ACTG1P4///AMY2B///AMY2A///AMY1C///AMY1B///AMY1A
13	ACTN1
14	ACTN4
15	ACVR1B
16	ACVR2A
17	ADA
18	ADAM19
19	ADAM9
20	ADCY3
21	ADD2
22	ADGRE2
23	ADGRE3
24	ADGRF3
25	ADPRM
26	ADRB2
27	ADTRP
28	AGFG1
29	AGFG2
30	AHCYL2
31	AHNAK
32	AHSA2
33	AIFM2
34	AK2
35	AK5
36	AKT3
37	ALAS2
38	ALCAM
39	ALDH1L2
40	ALG14
41	ALPL

42	ALYREF
43	AMIGO1
44	ANAPC16
45	ANKH
46	ANKHD1
47	ANKRD31
48	ANKRD36BP2
49	ANLN
50	ANP32E
51	ANXA1
52	ANXA11
53	ANXA2
54	ANXA2P2
55	ANXA4
56	ANXA5
57	APBA2
58	APITD1-CORT///APITD1
59	APOBEC3B
60	APOBEC3G
61	APOL2
62	APP
63	ARAP2
64	ARAP3
65	ARHGAP11A
66	ARHGAP18
67	ARHGAP42
68	ARHGEF2
69	ARHGEF40
70	ARID5B
71	ARL4C
72	ARL6IP1
73	ARMCX5-GPRASP2///GPRASP2
74	ARNTL2
75	ARPC5L
76	ARRB1
77	ARRDC3
78	ASC1P100
79	ASF1B
80	ASNS
81	ASPH
82	ASPM
83	ATAD2
84	ATF2
85	ATL2

86	ATM
87	ATP1B1
88	ATP1B3
89	ATP2A2
90	ATP2B4
91	ATP6AP1
92	ATP6AP2
93	ATXN1
94	AURKA
95	AURKB
96	AUTS2
97	AXL
98	B4GALT5
99	B9D1
100	BACH2
101	BAG1
102	BAG2
103	BAIAP2
104	BARD1
105	BAZ2A
106	BBS2
107	BCAM
108	BCAS4
109	BCAT1
110	BCL2
111	BCL2L11
112	BCL2L13
113	BEX2
114	BEX3
115	BEX5
116	BFSP1
117	BHLHA15
118	BHLHE40
119	BIRC5
120	BMI1
121	BMS1P20
122	BMS1P6
123	BRCA1
124	BRCA2
125	BRD2
126	BRD4
127	BRD8
128	BRI3BP
129	BRIP1
130	BTG3

131	BUB1
132	BUB1B
133	C10orf35
134	C11orf21
135	C12orf57
136	C12orf75
137	C14orf28
138	C17orf96
139	C17orf97
140	C19orf33
141	C1GALT1C1
142	C1orf106
143	C1orf123
144	C1orf162
145	C1orf56
146	C1QB
147	C21orf2
148	C2orf40
149	C3orf14
150	C3orf58
151	C4orf32
152	C4orf46
153	C5orf63
154	C6orf120
155	C6orf52
156	C9orf72
157	CA6
158	CACNA2D3
159	CADM1
160	CALR
161	CALU
162	CAND2
163	CARMIL1
164	CASKIN2
165	CASS4
166	CAV1
167	CBX1
168	CBY1
169	CCDC134
170	CCDC150
171	CCDC167
172	CCDC18-AS1
173	CCDC50
174	CCDC58
175	CCDC65

176	CCDC7
177	CCL28
178	CCL4
179	CCL5
180	CCNA2
181	CCNB1
182	CCNB2
183	CCND2
184	CCND3
185	CCNE1
186	CCNE2
187	CCNF
188	CCNJL
189	CCR1
190	CCR10
191	CCR2
192	CCR3
193	CCR5
194	CCR7
195	CD109
196	CD38
197	CD53
198	CD58
199	CD59
200	CD6
201	CD74
202	CD86
203	CD99
204	CDC123
205	CDC20
206	CDC25A
207	CDC37L1
208	CDC45
209	CDC6
210	CDC7
211	CDCA2
212	CDCA3
213	CDCA5
214	CDCA7
215	CDCA8
216	CDK1
217	CDK2
218	CDK2AP1
219	CDK4
220	CDK5R1

221	CDKN2A
222	CDKN2C
223	CDKN3
224	CDS2
225	CDT1
226	CEBPD
227	CENPA
228	CENPE
229	CENPF
230	CENPI
231	CENPM
232	CENPN
233	CENPQ
234	CENPU
235	CENPW
236	CEP104
237	CEP128
238	CEP152
239	CEP55
240	CEP68
241	CEP76
242	CES1
243	CFAP44
244	CFL2
245	CHAC2
246	CHAF1A
247	CHAF1B
248	CHCHD2
249	CHEK1
250	CHI3L1
251	CHID1
252	CHP1
253	CHRM3-AS2
254	CIITA
255	CISD2
256	CIZ1
257	CKAP2L
258	CKS1B
259	CKS2
260	CLDN1
261	CLEC11A
262	CLECL1
263	CLIC1
264	CLIC4
265	CLN5

266	CLNS1A
267	CLSPN
268	CLU
269	CLUHP3///ZNF720
270	CMPK2
271	CMTM2
272	CMTM7
273	CNN3
274	CNTN1
275	CNTNAP3
276	CNTNAP3B
277	COA1
278	COBLL1
279	COL10A1
280	COLGALT1
281	CORO1C
282	COTL1
283	COX8A
284	CPPED1
285	CPSF2
286	CR2
287	CREB5
288	CREBBP
289	CREM
290	CRIP1
291	CRTAM
292	CRTAP
293	CSGALNACT1
294	CSNK1A1
295	CSRP1
296	CST7
297	CTLA4
298	CTNNA1
299	CTNNAL1
300	CTSB
301	CTSC
302	CTSD
303	CTSL1
304	CX3CR1
305	CXCL1
306	CXCR3
307	CXCR6
308	CXorf26
309	CYAT1

310	CYAT1///IGLV1-44///IGLC1
311	CYAT1///IGLV1-44///IGLV7-43
312	CYP20A1
313	CYP24A1
314	CYP2R1
315	CYP3A5
316	CYP51A1
317	DCAF12
318	DCHS1
319	DCPS
320	DDIAS
321	DDR2
322	DDX3Y
323	DDX58
324	DENND1B
325	DENND2D
326	DENND5A
327	DENND5B
328	DEPDC1
329	DEPDC1B
330	DERL3
331	DESI2
332	DGKA
333	DHFR
334	DHRS1
335	DHRS3
336	DHX8
337	DIAPH3
338	DKC1
339	DKFZp781G1976
340	DKK3
341	DLEU2
342	DLG3
343	DLG5
344	DLGAP5
345	DMC1
346	DMTN
347	DNAJB11
348	DNAJB12
349	DNAJC3
350	DNAJC30
351	DOCK5
352	DOCK9

353	DONSON
354	DPP3
355	DSC1
356	DSC2
357	DSCC1
358	DSEL
359	DSP
360	DTHD1
361	DTL
362	DTWD1
363	DUSP10
364	DUSP4
365	DUSP5
366	DUT
367	DVL2
368	E2F1
369	E2F2
370	E2F7
371	E2F8
372	EAF2
373	ECI2
374	ECT2
375	EEF1A1
376	EEF1A2
377	EFCAB11
378	EFCAB13
379	EFHD2
380	EFR3A
381	EGR1
382	EGR3
383	EHD4
384	EIF1
385	EIF1AY
386	EIF3B
387	EIF3D
388	EIF3M
389	EIF4ENIF1
390	ELF3
391	ELL2
392	EME1
393	ENC1
394	ENDOG
395	EPB42
396	EPDR1
397	EPHA1-AS1

398	EPHB4
399	EPHX2
400	EPPK1
401	EPSTI1
402	EPT1
403	ERAP1
404	ERC1
405	ERCC1
406	ERI1
407	ERLEC1
408	ERMP1
409	ESCO2
410	ESPL1
411	ESYT2
412	EXO1
413	EZH2
414	F11R
415	F2R
416	F2RL1
417	F8
418	FABP5
419	FADS1
420	FADS2
421	FAHD1
422	FAM111B
423	FAM115A
424	FAM117B
425	FAM126A
426	FAM129A
427	FAM134B
428	FAM166A
429	FAM169A
430	FAM175A
431	FAM177A1
432	FAM184B
433	FAM185A
434	FAM207A
435	FAM208B
436	FAM210B
437	FAM212B
438	FAM223A///FAM223B
439	FAM226B///FAM226A
440	FAM3A
441	FAM46C
442	FAM49A

443	FAM72A///FAM72B///FAM72C
444	FAM72A///FAM72D///FAM72B///FAM72C
445	FAM83D
446	FAM98A
447	FANCG
448	FANCI
449	FANCL
450	FAS
451	FASLG
452	FBLN5
453	FBRSL1
454	FBXL4
455	FBXO31
456	FBXO32
457	FBXO5
458	FBXO6
459	FBXO7
460	FCER1A
461	FCGBP
462	FCGR3B
463	FCGR3B///FCGR3A
464	FCGRT
465	FCHO2
466	FCRL5
467	FDFT1
468	FEN1
469	FGF2
470	FGF9
471	FGFBP2
472	FH
473	FHIT
474	FKBP10
475	FKBP11
476	FLNA
477	FLNB
478	FNDC3A
479	FNIP2
480	FNTB
481	FOSL2
482	FOXK2
483	FOXM1
484	FOXO3
485	FOXO3B///FOXO3

486	FOXP1
487	FTH1
488	FTSJ1
489	FTSJD2
490	FTX
491	FXR1
492	GABBR1
493	GAL3ST4
494	GALM
495	GALNT1
496	GALNT2
497	GAPDH
498	GAS6
499	GATAD2B
500	GBP1
501	GCC2
502	GCNT1
503	GCNT4
504	GCSAM
505	GEN1
506	GFI1
507	GFOD1
508	GFPT1
509	GGH
510	GGTA1P
511	GIMAP1-GIMAP5///GIMAP5
512	GINS1
513	GINS2
514	GINS3
515	GINS4
516	GK5
517	GKAP1
518	GLB1
519	GLDC
520	GLUL
521	GMNN
522	GMPPB
523	GNAQ
524	GNAS
525	GNL3L
526	GNLY
527	GNPAT
528	GOLIM4
529	GOLM1

530	GON7
531	GP5
532	GPCPD1
533	GPKOW
534	GPM6B
535	GPR146
536	GPRASP1
537	GPRC5D
538	GPRIN3
539	GRAMD1A
540	GRAMD1C
541	GRIP1
542	GRWD1
543	GSG2
544	GSTM3
545	GTF2I
546	GTSE1
547	GUSBP11
548	GZMA
549	GZMB
550	GZMH
551	H2AFJ
552	H2AFX
553	H2BFS
554	HAUS1
555	HAVCR2
556	HBB
557	HBG2///HBG1
558	HBQ1
559	HCAR3
560	HCG8///ZNRD1ASP
561	HDAC8
562	HDLBP
563	HELLS
564	HERC5
565	HEY1
566	HIPK2
567	HIST1H1A
568	HIST1H1B
569	HIST1H1C
570	HIST1H1D
571	HIST1H2AB
572	HIST1H2AE
573	HIST1H2AJ
574	HIST1H2AL

575	HIST1H2AM
576	HIST1H2BB
577	HIST1H2BE
578	HIST1H2BH
579	HIST1H2BJ///HIST1H2BG
580	HIST1H2BM
581	HIST1H2BN
582	HIST1H2BO
583	HIST1H3B
584	HIST1H3C
585	HIST1H3D///HIST1H2AD
586	HIST1H3F
587	HIST1H3G
588	HIST1H3I
589	HIST1H4J
590	HIST1H4L
591	HIST3H2A
592	HIVEP3
593	HJURP
594	HLA-DMA
595	HLA-DOA
596	HLA-DPA1
597	HLA-DQA1
598	HLA-DRA
599	HLA-DRB1
600	HLA-DRB4
601	HLA-DRB6
602	HMGA1
603	HMGB2
604	HMGB3
605	HMGB3P1
606	HMMR
607	HMMR-AS1
608	HN1
609	HNRNPLL
610	HOOK1
611	HOPX
612	HRASLS
613	HRASLS2
614	HSD17B4
615	HSP90B1
616	HSPA13
617	HSPA5
618	HSPB11
619	HSPC102

620	HSPD1
621	HUWE1
622	HYOU1
623	ICA1
624	IDE
625	IDH2
626	IFI27
627	IFI27L1
628	IFI44L
629	IFIT1
630	IFIT2
631	IFIT3
632	IFNG
633	IFNLR1
634	IGF1
635	IGF1R
636	IGF2BP2
637	IGHD
638	IGHG1///IGHD///IGHA2///IGH
639	IGHM
640	IGHM///IGHG4///IGHG3///IGHG1///IGHD///IGHA2///IGHA1///IGH
641	IGHV3-47
642	IGHV4-31///IGHM///IGHG3///IGHG1///IGHA2///IGHA1///IGH
643	IGHV4-31///IGHM///IGHG4///IGHG3///IGHG1///IGHD///IGHA2///IGHA1///IGH
644	IGIP
645	IGKV1D-13
646	IGKV3-20
647	IGKV4-1
648	IGL
649	IGLJ3
650	IGLJ3///CKAP2///IGLV@///IGLC1
651	IGLJ3///IGLV1-44///CKAP2///IGLV@///IGLC1
652	IGLL3P

653	IGLL3P///GUSBP11///IGLL1
654	IGLL5///IGLV3-16///IGLV3-25///IGLC1
655	IGLV1-44
656	IGLV2-23
657	IGLV3-19
658	IGLV4-60
659	IGLV6-57
660	IL12RB2
661	IL13RA1
662	IL18RAP
663	IL1B
664	IL1RAP
665	IL23A
666	IL24
667	IL2RB
668	IL6R
669	IL6ST
670	IL7R
671	INPP5K
672	IREB2
673	IRF2BP2
674	IRF4
675	IRS2
676	ISG15
677	ITFG3
678	ITGA6
679	ITGAM
680	ITGB1
681	ITGB4
682	ITGB5
683	ITLN1
684	ITM2C
685	ITPKB
686	ITPR3
687	ITPRIP
688	ITPRIPL2
689	JAKMIP1
690	JAZF1
691	JUN
692	KANSL1
693	KANSL1L
694	KATNAL1
695	KATNBL1

696	KAZN
697	KBTBD8
698	KCNJ2
699	KCNN3
700	KCTD1
701	KCTD14
702	KDM1A
703	KDM4B
704	KDM5C
705	KDM5D
706	KEAP1
707	KIAA0101
708	KIAA0141
709	KIAA0355
710	KIAA1147
711	KIAA1324
712	KIAA1522
713	KIAA1671
714	KIF11
715	KIF14
716	KIF15
717	KIF18A
718	KIF18B
719	KIF20A
720	KIF20B
721	KIF22
722	KIF23
723	KIF2C
724	KIF4A
725	KIFC1
726	KLB
727	KLF16
728	KLF2
729	KLF7
730	KLF9
731	KLHDC1
732	KLHL14
733	KLHL3
734	KLHL34
735	KLHL9
736	KLRC4
737	KLRD1
738	KNL1
739	KPNA2
740	KPNA5

741	KRT1
742	KRT23
743	KRT6B
744	KTN1
745	LAG3
746	LAMC1
747	LAP3
748	LAPTM4B
749	LARP4
750	LARP7
751	LCP2
752	LDLR
753	LDLRAP1
754	LEF1
755	LEF1-AS1
756	LEPREL2
757	LEPROT
758	LGALS1
759	LGALS3
760	LGALS3BP
761	LGALSL
762	LGMN
763	LHX4
764	LIM2
765	LIMK2
766	LIMS4///LIMS3///LIMS1
767	LINC00282
768	LINC00467
769	LINC00663
770	LINC00954
771	LINC01128
772	LINC01550
773	LIPA
774	LMF1
775	LMLN
776	LMNB1
777	LMO7
778	LOC100130872///SPON2
779	LOC100131541
780	LOC100132910///GALNT2
781	LOC100133862///IGHM///IGHG1
782	LOC100287723
783	LOC100287896

784	LOC100289230
785	LOC100289612
786	LOC100291464///IGK///IGKC
787	LOC100291464///LOC100130100
788	LOC100291682///IGK///IGKV3-20///IGKC
789	LOC100292999///LOC100290059///IGHD
790	LOC100293277///IGLC1
791	LOC100293440
792	LOC100293440///IGLV2-23
793	LOC100505501
794	LOC100505715
795	LOC100506548///RPL37
796	LOC100506990
797	LOC100996286
798	LOC100996385
799	LOC100996809///HLA-DRB4///HLA-DRB1
800	LOC101060391
801	LOC101060835///LOC100996809///HLA-DRB4///HLA-DRB3///HLA-DRB1///HLA-DQB1
802	LOC101060835///LOC100996809///HLA-DRB5///HLA-DRB4///HLA-DRB1///HLA-DQB1
803	LOC101927402
804	LOC101928140
805	LOC101928173
806	LOC101928893
807	LOC101929373///SLC9B1
808	LOC101930363///LOC101928349///LOC100507387///FAM153C///FAM153A///FAM153B
809	LOC101930370
810	LOC101930489///MIR4435-2HG///LINC00152

811	LOC102606465
812	LOC102725213///LOC100507387///FAM153A///FAM153B
813	LOC105372881
814	LOC107985971
815	LOC202025
816	LOC286052
817	LOC652493
818	LOC652493///IGK///IGKC
819	LOC652494///IGHM
820	LOC652494///IGHV4-31///IGHM///IGHG3///IGHG1///IGHA1
821	LOC727820
822	LOC730101
823	LONP2
824	LPCAT3
825	LRIG2
826	LRP8
827	LRR1
828	LRRC59
829	LRRN3
830	LSS
831	LUC7L
832	LY9
833	LYZ
834	MAD2L1
835	MAD2L1BP
836	MAF
837	MAGED2
838	MAGEE1
839	MAGI3
840	MAGT1
841	MAK
842	MAL
843	MAML2
844	MAN1A1
845	MAN1C1
846	MANEA
847	MANF
848	MAP2K6
849	MAP3K5
850	MAP3K7CL

851	MAP4K4
852	MAP7
853	MAST4
854	MASTL
855	MATN1-AS1
856	MAVS
857	MB21D1
858	MBNL3
859	MCC
860	MCM10
861	MCM2
862	MCM4
863	MCM5
864	MCM6
865	MCM7
866	MCOLN2
867	MCUR1
868	MED12
869	MED14
870	MED18
871	MED31
872	MEGF6
873	MELK
874	MEST
875	MET
876	MFN1
877	MFSD8
878	MGA
879	MGAT4A
880	MGC16275
881	MGME1
882	MGRN1
883	MICAL2
884	MICB
885	MID1
886	MID1IP1
887	MID2
888	MINPP1
889	MIR1282///HYPK///SERF2
890	MIR15A///DLEU2
891	MIR15A///DLEU2L///DLEU2
892	MIR21///VMP1
893	MIR3658///UCK2

894	MIR4435-2HG
895	MIR6845///NRBP2
896	MIR6883///PER1
897	MKI67
898	MLH3
899	MLLT3
900	MLXIP
901	MMADHC
902	MMD
903	MMP28
904	MND1
905	MOGS
906	MORF4L1
907	MORF4L2
908	MPC2
909	MPP7
910	MRPL38
911	MRPS18C
912	MT1E
913	MT2A
914	MTERF2
915	MTFR2
916	MTHFD1
917	MTHFD1L
918	MTHFD2
919	MTUS1
920	MX1
921	MYBL2
922	MYDGF
923	MYL4
924	MYL6B
925	MYO1D
926	MYO1F
927	MZB1
928	N/A
929	N4BP2L2
930	NAAA
931	NADK
932	NBPF1
933	NBPF20///NBPF10///NBPF8///NBPF9///NBPF15///NBPF12///NBPF14

934	NBPF25P///NBPF26///NBPF19///NBPF20///NBPF10///NBPF8///NBPF9///NBPF15///NBPF11///NBPF14
935	NCAPG
936	NCAPG2
937	NCAPH
938	NCOA3
939	NCOR1
940	NDC1
941	NDC80
942	NDFIP1
943	NDUFA8
944	NDUFB11
945	NDUFS5
946	NEFH
947	NEIL3
948	NEK2
949	NELL2
950	NET1
951	NETO2
952	NFIA
953	NFIL3
954	NFKBIZ
955	NFYC-AS1
956	NHS
957	NKG7
958	NME1
959	NOG
960	NOL4L
961	NOSIP
962	NOV
963	NOVA1
964	NPAS2
965	NR1D1///THRA
966	NR3C2
967	NRCAM
968	NREP
969	NRG1
970	NRM
971	NRP1
972	NSG1
973	NSUN6
974	NSUN7

975	NT5C1B-RDH14///NT5C1B
976	NT5DC2
977	NT5DC4
978	NT5E
979	NTPCR
980	NUAK2///AKIP1
981	NUCB1
982	NUF2
983	NUMA1
984	NUP107
985	NUP188
986	NUP210
987	NUP35
988	NUP50
989	NUSAP1
990	NVL
991	O95081
992	OAS1
993	OASL
994	OGN
995	OIP5
996	OLIG1
997	OR2W3
998	ORC1
999	ORC6
1000	OSBP2
1001	OSBPL3
1002	OSER1-AS1
1003	OSGEP
1004	OTUD7A
1005	OXNAD1
1006	P2RY1
1007	P4HA2
1008	P4HB
1009	PABPC1
1010	PABPC4
1011	PACRGL
1012	PACSIN1
1013	PAICS
1014	PAIP1
1015	PALD1
1016	PALM2-AKAP2///AKAP2
1017	PAM
1018	PARM1

1019	PARPBP
1020	PARVB
1021	PASK
1022	PATJ
1023	PBK
1024	PCCB
1025	PCMTD1
1026	PCNA
1027	PCSK5
1028	PDCD4-AS1
1029	PDE3B
1030	PDE7A
1031	PDE9A
1032	PDIA3
1033	PDIA4
1034	PDIA5
1035	PDIA6
1036	PDK1
1037	PDZK1IP1
1038	PEG10
1039	PELI2
1040	PERP
1041	PEX19
1042	PFKFB3
1043	PFN2
1044	PGGHG
1045	PHACTR2
1046	PHC3
1047	PHF19
1048	PHGDH
1049	PHLDA1
1050	PHTF1
1051	PI3
1052	PIGS
1053	PIK3AP1
1054	PIK3IP1
1055	PIP4K2A
1056	PITPNB
1057	PKHD1L1
1058	PLA2G12A
1059	PLAG1
1060	PLCG2
1061	PLEK
1062	PLEKHA1
1063	PLEKHB1

1064	PLK1
1065	PLK4
1066	PLOD1
1067	PLOD2
1068	PLP2
1069	PLPP6
1070	PLS3
1071	PLSCR1
1072	PLXDC1
1073	PMAIP1
1074	PMCH
1075	PMCHL1
1076	PMF1
1077	PMM2
1078	PNISR
1079	PNO1
1080	POC1A
1081	POLA1
1082	POLE2
1083	POLI
1084	POLQ
1085	POLR2D
1086	POMP
1087	PON2
1088	POU2AF1
1089	PPBP
1090	PPIB
1091	PPIF
1092	PPOX
1093	PPP1CA
1094	PPP1R3E
1095	PPP4R2
1096	PRAF2
1097	PRC1
1098	PRDM1
1099	PRDX3
1100	PRDX4
1101	PRF1
1102	PRKAG2-AS1
1103	PRKCA
1104	PRKCA-AS1
1105	PRKCQ-AS1
1106	PRKRIP1
1107	PRKXP1
1108	PRMT2

1109	PRPF31
1110	PRPF39
1111	PRR11
1112	PRR12
1113	PRSS33
1114	PSAP
1115	PSAT1
1116	PSMA1
1117	PSMB9
1118	PSMG4
1119	PSPH
1120	PSTPIP2
1121	PTCD3
1122	PTCH1
1123	PTGS2
1124	PTMS
1125	PTPRF
1126	PTPRJ
1127	PTPRK
1128	PTPRN2
1129	PTTG1
1130	PTTG3P
1131	PURA
1132	PWAR5
1133	PWAR6
1134	PWP1
1135	PXYLP1
1136	PYHIN1
1137	R3HDM4
1138	RAB11A
1139	RAB11FIP1
1140	RAB27A
1141	RAB30
1142	RAB8B
1143	RABAC1
1144	RACGAP1
1145	RAD51
1146	RAD51AP1
1147	RALGAPB
1148	RALGPS2
1149	RANBP1
1150	RANBP2
1151	RAP1GAP2
1152	RAPGEF6
1153	RASGEF1A

1154	RASGRF2
1155	RBBP7
1156	RBBP8
1157	RBL2
1158	RBM22
1159	RBM26
1160	RBM28
1161	RBM42
1162	RBM7
1163	RBPMS
1164	RCAN3
1165	RECQL4
1166	REEP4
1167	REEP5
1168	REG4
1169	RELA
1170	REPS2
1171	RETSAT
1172	RFC3
1173	RFC5
1174	RFX2
1175	RGCC
1176	RGMB
1177	RGS1
1178	RGS16
1179	RHOU
1180	RIMKLB
1181	RMI2
1182	RNASE1
1183	RNASEH2A
1184	RNF135
1185	RNF144A
1186	RNF157-AS1
1187	ROBO1
1188	ROBO3
1189	RPGRIP1
1190	RPL15
1191	RPL17-C18orf32///SNORD58C///SNORD58A///SNORD58B///RPL17
1192	RPL22
1193	RPL31
1194	RPL37A
1195	RPS23

1196	RPS4Y1
1197	RRBP1
1198	RRM1
1199	RRM2
1200	RRP12
1201	RRP36
1202	RTN3
1203	RTN4
1204	RTN4IP1
1205	RUNDC3A
1206	RUNX2
1207	S100A11
1208	SAMSN1
1209	SAP30
1210	SAR1B
1211	SATB1
1212	SATB1-AS1
1213	SCAF11
1214	SCARNA13///SNHG10
1215	SCD
1216	SCD5
1217	SCIN
1218	SCML1
1219	SDC1
1220	SDC2
1221	SDCCAG8
1222	SDF2
1223	SDF2L1
1224	SEC11C
1225	SEC14L1
1226	SEC16
1227	SEC24A
1228	SEC24D
1229	SEC31B
1230	SEC61A1
1231	SEL1L3
1232	SELENBP1
1233	SEPHS1
1234	SESN3
1235	SETBP1
1236	SF1
1237	SFSWAP
1238	SFXN2
1239	SGK223
1240	SGO2

1241	SGSM2
1242	SGTA
1243	SH3BGRL2
1244	SH3YL1
1245	SHCBP1
1246	SHKBP1
1247	SHMT1
1248	SHMT2
1249	SIAE
1250	SIGLEC1
1251	SIGLEC12///SIGLEC10
1252	SIGLEC17P
1253	SIK1
1254	SKA1
1255	SKA2
1256	SKA3
1257	SLAMF1
1258	SLAMF7
1259	SLBP
1260	SLC12A2
1261	SLC14A1
1262	SLC16A1
1263	SLC16A10
1264	SLC16A14
1265	SLC1A4
1266	SLC1A5
1267	SLC22A23
1268	SLC25A25-AS1
1269	SLC25A37
1270	SLC25A39
1271	SLC26A11
1272	SLC27A2
1273	SLC2A5
1274	SLC35F6///CENPA
1275	SLC38A10
1276	SLC3A2
1277	SLC40A1
1278	SLC43A3
1279	SLC44A1
1280	SLC45A4
1281	SLC4A1
1282	SLC6A8
1283	SLC7A5
1284	SLC7A6
1285	SLC7A8

1286	SLCO4A1
1287	SLMAP
1288	SLPI
1289	SMAD3
1290	SMC2
1291	SMC4
1292	SMCO4
1293	SMIM14
1294	SMIM24
1295	SMIM8
1296	SMNP
1297	SMS
1298	SNCA
1299	SNORD50B///SNORD50A
1300	SNORD68///RPL13
1301	SNRPE
1302	SNX10
1303	SNX5
1304	SNX9
1305	SORL1
1306	SOX4
1307	SP3
1308	SPAG5
1309	SPATA13
1310	SPATS2
1311	SPATS2L
1312	SPC24
1313	SPC25
1314	SPCS3
1315	SPEF2
1316	SPG20
1317	SPIN3
1318	SPIN4
1319	SPINK2
1320	SPINT2
1321	SPSB1
1322	SPTLC1
1323	SPTLC2
1324	SPTSSA
1325	SRGN
1326	SRP19
1327	SRP54
1328	SRP68
1329	SRP72

1330	SRPR
1331	SRPRB
1332	SSBP2
1333	SSR1
1334	SSR3
1335	SSR4
1336	STIL
1337	STIM1
1338	STK39
1339	STK4
1340	STMN1
1341	STMN2
1342	STMN3
1343	STOM
1344	STRADB
1345	STRBP
1346	STT3A
1347	STT3B
1348	STX11
1349	STX3
1350	STXBP3
1351	SULT1B1
1352	SUPT3H
1353	SUSD1
1354	SUV39H2
1355	SUZ12
1356	SVIL
1357	SVIP
1358	SYMPK
1359	SYNE1
1360	SYNE2
1361	SYNJ2BP-COX16///SYNJ2BP
1362	SYT11
1363	TACC3
1364	TAL1
1365	TALDO1
1366	TAOK2
1367	TARP///TRGV9///TRGC2
1368	TBC1D10A
1369	TBK1
1370	TBL2
1371	TBX21
1372	TCEA3
1373	TCEAL2

1374	TCEAL3
1375	TCEAL4
1376	TCF19
1377	TCF7
1378	TCP11L2
1379	TESC
1380	TFDP1
1381	TGFBI
1382	TGFBR3
1383	TGIF1
1384	TGM2
1385	TGOLN2
1386	THBD
1387	THEM4
1388	THNSL1
1389	TIGD1
1390	TIGD3
1391	TIGIT
1392	TIMELESS
1393	TIPIN
1394	TJP2
1395	TK1
1396	TMEM106C
1397	TMEM133
1398	TMEM161B-AS1
1399	TMEM165
1400	TMEM2
1401	TMEM30B
1402	TMEM33
1403	TMEM50B
1404	TMEM97
1405	TMOD1
1406	TMOD2
1407	TMPO
1408	TMPRSS3
1409	TMTC3
1410	TMX2
1411	TMX4
1412	TNFRSF10C
1413	TNFRSF17
1414	TNFRSF1B
1415	TNFSF8
1416	TNIP3
1417	TNRC6B
1418	TNRC6C

1419	TNRC6C-AS1
1420	TNS1
1421	TOB1
1422	TOP2A
1423	TOR1B
1424	TOR4A
1425	TOX
1426	TP53INP1
1427	TP73-AS1
1428	TPM2
1429	TPM4
1430	TPX2
1431	TRA2B
1432	TRABD2A
1433	TRAF3IP3
1434	TRAM2
1435	TRAPPC13
1436	TREM1
1437	TRIB1
1438	TRIM28
1439	TRIM59
1440	TRIP13
1441	TRMT1L
1442	TROAP
1443	TRPC1
1444	TRPM6
1445	TSC22D3
1446	TSHR
1447	TSPAN2
1448	TSPAN5
1449	TSPYL1
1450	TTC28
1451	TTC39B
1452	TTC3P1///TTC3
1453	TTC9
1454	TTK
1455	TTN
1456	TTN-AS1
1457	TUBA1B
1458	TUBA1C
1459	TUBB
1460	TUBB2A
1461	TUBB3
1462	TUBB4B
1463	TUBE1

1464	TUBG1
1465	TXK
1466	TXLNGY
1467	TXN
1468	TXNDC11
1469	TXNDC17
1470	TXNDC5///BLOC1S5
1471	TXNIP
1472	TYMS
1473	TYSND1
1474	UAP1
1475	UBA5
1476	UBE2C
1477	UBE2F
1478	UBE2J1
1479	UBE2S
1480	UBE2T
1481	UBN2
1482	UBTF
1483	UBXN7
1484	UCHL1
1485	UCK2
1486	UHRF1
1487	USO1
1488	USP18
1489	USP28
1490	USP34
1491	USP51
1492	USP53
1493	UTS2
1494	VIMP
1495	VMA21
1496	VMP1
1497	VSIG1
1498	VWA5A
1499	WAC
1500	WARS
1501	WBP11
1502	WBP7
1503	WDFY2
1504	WDHD1
1505	WDR34
1506	WDR37
1507	WDR76
1508	WEE1

1509	WFS1
1510	WHAMMP2
1511	WHAMMP2///WHAMMP3
1512	WHSC1
1513	WLS
1514	XIST
1515	XRCC1
1516	YBX3
1517	YIF1B
1518	YLPM1
1519	YPEL2
1520	YWHAE
1521	YWHAH
1522	ZBED1
1523	ZBP1
1524	ZBTB10
1525	ZBTB18
1526	ZBTB20
1527	ZC3H6
1528	ZC3H8
1529	ZC3HAV1
1530	ZC3HAV1L
1531	ZCCHC10
1532	ZCCHC8
1533	ZDHHC2
1534	ZEB1
1535	ZEB2
1536	ZFAND6
1537	ZFP28
1538	ZFP91
1539	ZMAT1
1540	ZMYM3
1541	ZNF217
1542	ZNF24
1543	ZNF260
1544	ZNF264
1545	ZNF302
1546	ZNF329
1547	ZNF337
1548	ZNF347
1549	ZNF367
1550	ZNF397
1551	ZNF439
1552	ZNF440

1553	ZNF483
1554	ZNF493
1555	ZNF506
1556	ZNF548
1557	ZNF550
1558	ZNF558
1559	ZNF559
1560	ZNF573
1561	ZNF577
1562	ZNF585A
1563	ZNF595
1564	ZNF609
1565	ZNF629
1566	ZNF641
1567	ZNF667-AS1
1568	ZNF677
1569	ZNF683
1570	ZNF711
1571	ZNF717
1572	ZNF776
1573	ZNF780B
1574	ZNF829
1575	ZNF84
1576	ZNF844
1577	ZNF862
1578	ZNF91
1579	ZNRD1ASP
1580	ZSCAN18
1581	ZWILCH
1582	ZWINT
1583	ZXDB
1584	ZZZ3

Table S2 Drug candidates identified using CMap tool

Sr. No.	**Name**	**Description**
1	JAK3-inhibitor-VI	JAK inhibitor
2	PPIE	RNA binding motif (RRM) containing
3	CD-437	Retinoid receptor agonist
4	ID4	Basic helix-loop-helix proteins
5	crizotinib	ALK inhibitor
6	CBS	Hydrogen sulphide synthesis
7	triclosan	Enoyl-[acyl-carrier-protein] reductase [NADH] inhibitor

8	PAN2	Ubiquitin-specific peptidases
9	ARHGEF7	Rho guanine nucleotide exchange factors
10	HIST2H2BE	Histones / Replication-dependent
11	A-443644	AKT inhibitor
12	canertinib	EGFR inhibitor
13	masitinib	KIT inhibitor
14	JNJ-7706621	CDK inhibitor
15	IKK-2-inhibitor-V	IKK inhibitor
16	obatoclax	BCL inhibitor
17	staurosporine	PKC inhibitor
18	SCH-79797	Proteasome inhibitor
19	niclosamide	DNA replication inhibitor
20	ryuvidine	Histone lysine methyltransferase inhibitor
21	sorafenib	FLT3 inhibitor
22	daunorubicin	RNA synthesis inhibitor
23	wortmannin	PI3K inhibitor
24	triptolide	RNA polymerase inhibitor
25	arctigenin	MEK inhibitor
26	elvitegravir	HIV integrase inhibitor
27	UK-356618	Metalloproteinase inhibitor
28	lopinavir	HIV protease inhibitor
29	VX-222	HCV inhibitor
30	emetine	Protein synthesis inhibitor
31	cephaeline	Protein synthesis inhibitor
32	narciclasine	Coflilin signaling pathway activator
33	homoharringtonine	Protein synthesis inhibitor
34	BCL2-inhibitor	BCL inhibitor
35	chlorpromazine	Dopamine receptor antagonist
36	panobinostat	HDAC inhibitor
37	temsirolimus	MTOR inhibitor
38	doxorubicin	Topoisomerase inhibitor
39	roscovitine	CDK inhibitor
40	rucaparib	PARP inhibitor
41	verrucarin-a	Protein synthesis inhibitor
42	lestaurtinib	FLT3 inhibitor
43	teniposide	Topoisomerase inhibitor
44	ellipticine	Topoisomerase inhibitor
45	SN-38	Topoisomerase inhibitor
46	bemesetron	Serotonin receptor antagonist
47	VX-702	p38 MAPK inhibitor
48	deoxycholic-acid	G protein-coupled receptor agonist
49	anisomycin	DNA synthesis inhibitor
50	L-689560	Glutamate receptor antagonist
51	chlordiazepoxide	Benzodiazepine receptor agonist
52	SPDEF	ETS Transcription Factors

53	VX-745	p38 MAPK inhibitor
54	NTNCB	Neuropeptide receptor antagonist
55	carbenoxolone	11-beta-HSD1 inhibitor
56	oligomycin-a	ATP synthase inhibitor
57	esculin	Antioxidant
58	aminoglutethimide	Glucocorticoid receptor antagonist
59	ethisterone	Progestogen hormone
60	TW-37	BCL inhibitor
61	PD-102807	Acetylcholine receptor antagonist
62	strophanthidin	ATPase inhibitor
63	AG-879	Angiogenesis inhibitor
64	SR-59230A	Adrenergic receptor antagonist
65	ZM-447439	Aurora kinase inhibitor
66	indirubin	CDK inhibitor
67	HDAC2	Histone deacetylases
68	SA-792728	Sphingosine kinase inhibitor
69	butoconazole	Bacterial cell wall synthesis inhibitor
70	phenylbutazone	Cyclooxygenase inhibitor
71	tyrphostin-A9	Protein tyrosine kinase inhibitor
72	CGP-71683	Neuropeptide receptor antagonist
73	rimexolone	Glucocorticoid receptor agonist
74	AR-A014418	Glycogen synthase kinase inhibitor
75	lamotrigine	Serotonin receptor antagonist
76	sappanone-a	Tyrosinase inhibitor
77	APOC2	Apolipoproteins
78	PRL-3-inhibitor-I	Tyrosine phosphatase inhibitor
79	ZSTK-474	PI3K inhibitor
80	zibotentan	Endothelin receptor antagonist
81	GARS	Aminoacyl tRNA synthetases / Class II
82	RO-16-6941	Monoamine oxidase inhibitor
83	KARS	Aminoacyl tRNA synthetases / Class II
84	ADAM10	ADAM metallopeptidase domain containing
85	lumicolchicine	Colchicine isomer, non-binder of microtubules
86	VU-0418946-1	HIF modulator
87	CREB3L4	basic leucine zipper proteins
88	SKF-89976A	GABA uptake inhibitor
89	AHCY	Adenosine turnover
90	MK-2206	AKT inhibitor
91	tyrphostin-AG-126	ERK1 and ERK2 phosphorylation inhibitor
92	PLD1	Phosphatidylcholine-specific phospholipase D
93	methylene-blue	Guanylyl cyclase inhibitor
94	GRWD1	WD repeat domain containing
95	HMGCS1	Lanosterol biosynthesis pathway
96	SB-203580	p38 MAPK inhibitor
97	GGPS1	Lanosterol biosynthesis pathway

98	5-<br>nonyloxytryptamine	Serotonin receptor agonist
99	USP47	Ubiquitin-specific peptidases
100	procainamide	Sodium channel blocker
101	NNC-55-0396	T-type calcium channel blocker
102	SKP2	F-boxes / Leucine-rich repeats
103	ZNF114	Zinc fingers, C2H2-type
104	DYNLL2	Cytoplasmic dyneins
105	DL-PDMP	Glucosyltransferase inhibitor
106	HLI-373	MDM inhibitor
107	WDR61	WD repeat domain containing
108	EIF4G1	Parkinson disease
109	PQ-401	IGF-1 inhibitor
110	clomipramine	Serotonin transporter inhibitor (SERT)
111	CHEMBL-374350	NFkB pathway inhibitor
112	TSPAN8	Tetraspanins
113	terfenadine	Histamine receptor antagonist
114	IL20	Interleukins and interleukin receptors
115	KU-55933	ATM kinase inhibitor
116	PHA-793887	CDK inhibitor
117	perhexiline	Carnitine palmitoyltransferase inhibitor
118	zosuquidar	P-glycoprotein inhibitor
119	pifithrin	Interleukin receptor antagonist
120	danusertib	Aurora kinase inhibitor
121	VU-0400193-3	Glutamate receptor modulator
122	tyrphostin-AG-1478	EGFR inhibitor
123	nefazodone	Adrenergic inhibitor
124	JNK-9L	JNK inhibitor
125	ICI-199441	Opioid receptor agonist
126	ispinesib	Kinesin-like spindle protein inhibitor
127	PTB1	AMPK activator
128	CGP-60474	CDK inhibitor
129	bicuculline	GABA receptor antagonist
130	INPP1	Inositol polyphosphate phosphatases
131	WZ-4002	EGFR inhibitor
132	remoxipride	Dopamine receptor antagonist
133	fasudil	Rho associated kinase inhibitor
134	HRH1	Histamine receptors
135	SID-26681509	Cathepsin inhibitor
136	OSI-027	MTOR inhibitor
137	AG-494	EGFR inhibitor
138	HDAC3	Histone deacetylases
139	benazepril	ACE inhibitor
140	tyrphostin-AG-112	Protein tyrosine kinase inhibitor
141	ENMD-2076	FLT3 inhibitor

142	SIRT2	Histone deacetylases
143	protriptyline	Tricyclic antidepressant
144	brucine	Glycine receptor antagonist
145	butein	EGFR inhibitor
146	palbociclib	CDK inhibitor
147	SIRT6	Histone deacetylases (HDACs)
148	PTPN1	Protein tyrosine phosphatases
149	BAY-59-3074	Cannabinoid receptor partial agonist
150	SA-792709	Retinoid receptor agonist
151	trioxsalen	DNA synthesis inhibitor
152	vanoxerine	Dopamine uptake inhibitor
153	STO-609	Calmodulin antagonist
154	WH-4023	SRC inhibitor
155	angiogenesis-inhibitor	Angiogenesis inhibitor
156	KAT6B	Histone acetyltransferases (HATs)
157	dorsomorphin	AMPK inhibitor
158	HG-5-113-01	Protein kinase inhibitor
159	IKK-16	IKK inhibitor
160	NSC-3852	HDAC inhibitor
161	ZG-10	JNK inhibitor
162	CGP-53353	EGFR inhibitor
163	menadione	Mitochondrial DNA polymerase inhibitor
164	valdecoxib	Cyclooxygenase inhibitor
165	BAX-channel-blocker	Cytochrome C release inhibitor
166	XL-147	PI3K inhibitor
167	lypressin	Vasopressin receptor agonist
168	papaverine	Phosphodiesterase inhibitor
169	aminopurvalanol-a	Tyrosine kinase inhibitor
170	nonoxynol-9	Membrane integrity inhibitor
171	penfluridol	T-type calcium channel blocker
172	BMP4	Bone morphogenetic proteins
173	barasertib	Aurora kinase inhibitor
174	CALM3	EF-hand domain containing
175	malonoben	Protein tyrosine kinase inhibitor
176	WZ-4-145	EGFR inhibitor
177	amperozide	Dopamine receptor antagonist
178	alisertib	Aurora kinase inhibitor
179	diphenyleneiodonium	Nitric oxide synthase inhibitor
180	tamibarotene	Retinoid receptor agonist
181	NVP-TAE684	ALK inhibitor
182	CGK-733	ATR kinase inhibitor
183	quinoclamine	Algicide
184	apicidin	HDAC inhibitor

185	PIK-75	DNA protein kinase inhibitor
186	NPI-2358	Tubulin inhibitor
187	BX-795	IKK inhibitor
188	GW-501516	PPAR receptor agonist
189	alvocidib	CDK inhibitor
190	AURKB	Aurora kinase
191	mammea-a	other antibiotic
192	thiostrepton	FOXM1 inhibitor
193	chloroxine	Opioid receptor antagonist
194	BIBX-1382	EGFR inhibitor
195	AG-14361	PARP inhibitor
196	rottlerin	MAP kinase inhibitor
197	alvespimycin	HSP inhibitor
198	piceatannol	SYK inhibitor
199	flutamide	Androgen receptor antagonist
200	BMS-536924	IGF-1 inhibitor
