# Supplementary figures and images for: A Transcriptomics-Based Bioinformatics Approach for Identification and In Vitro Screening of FDA-Approved Drugs for Repurposing against Dengue Virus-2
Source: Viruses. 2022 Sep 29;14(10):2150. doi: 10.3390/v14102150 (PMC9609047; doi:10.3390/v14102150)

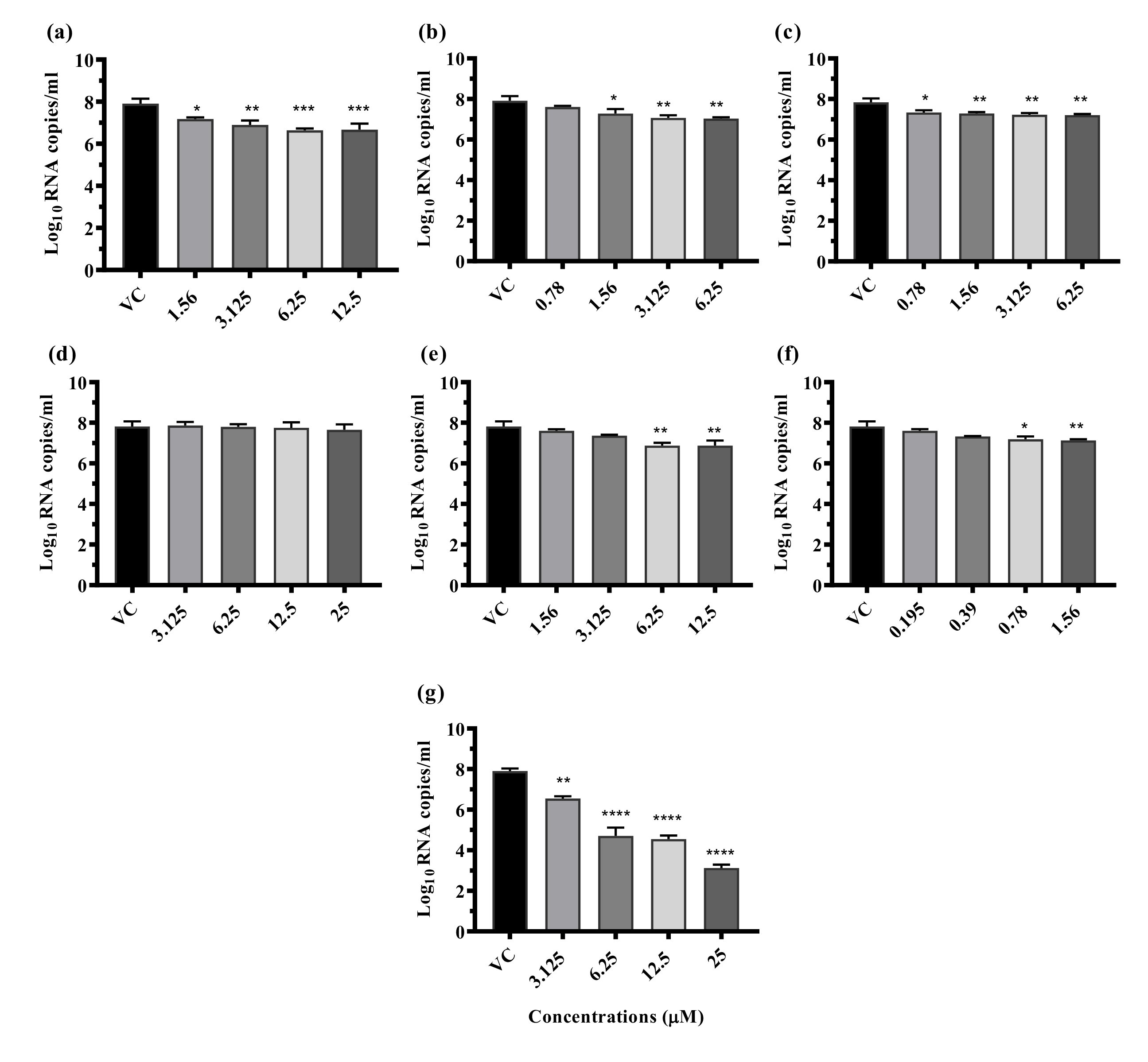

Supplement: Supplementary file 1 [file viruses-14-02150-s001.zip › Fig S2.tiff]

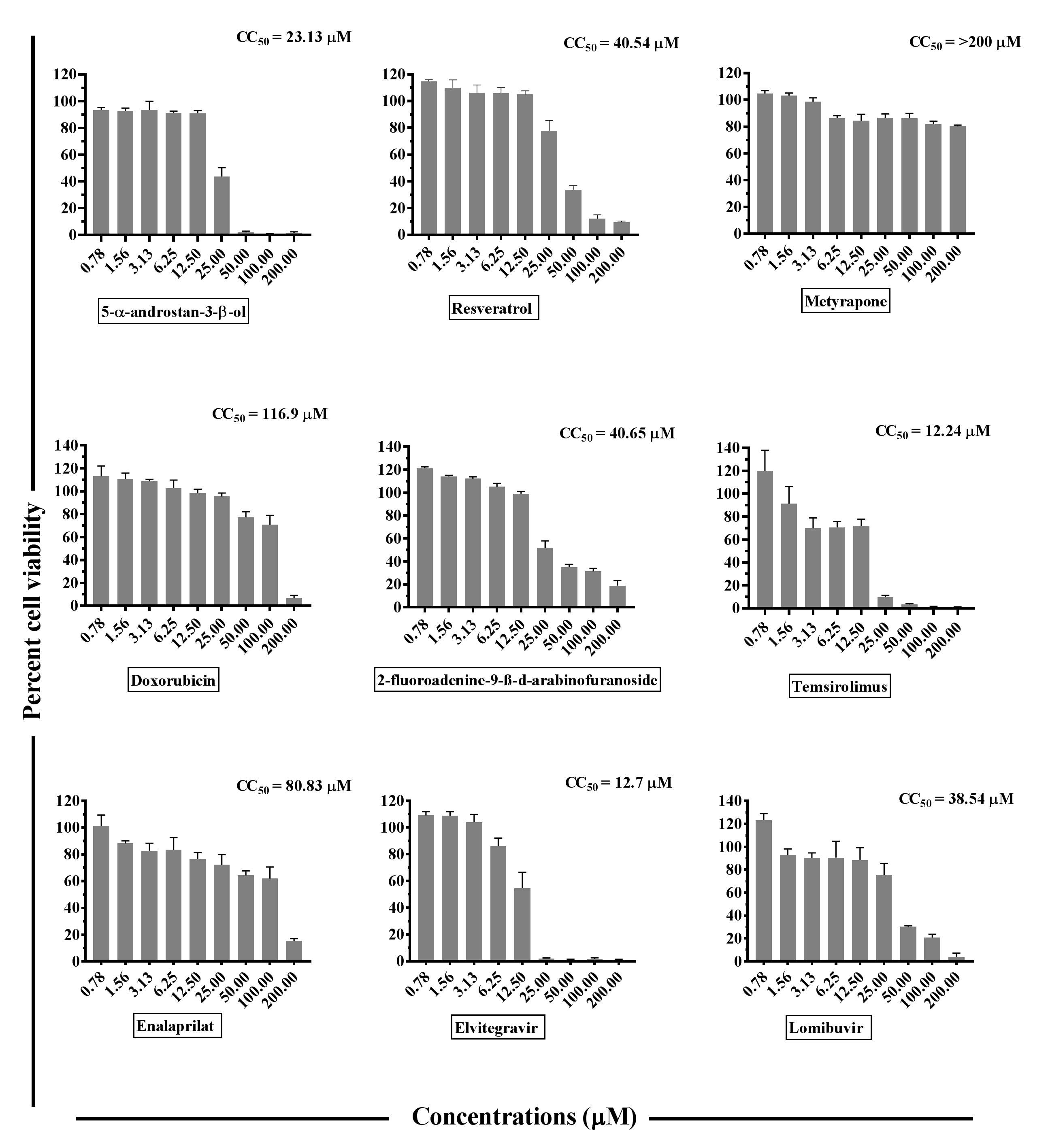

Supplement: Supplementary file 1 [file viruses-14-02150-s001.zip › Figure S1_1.tif]

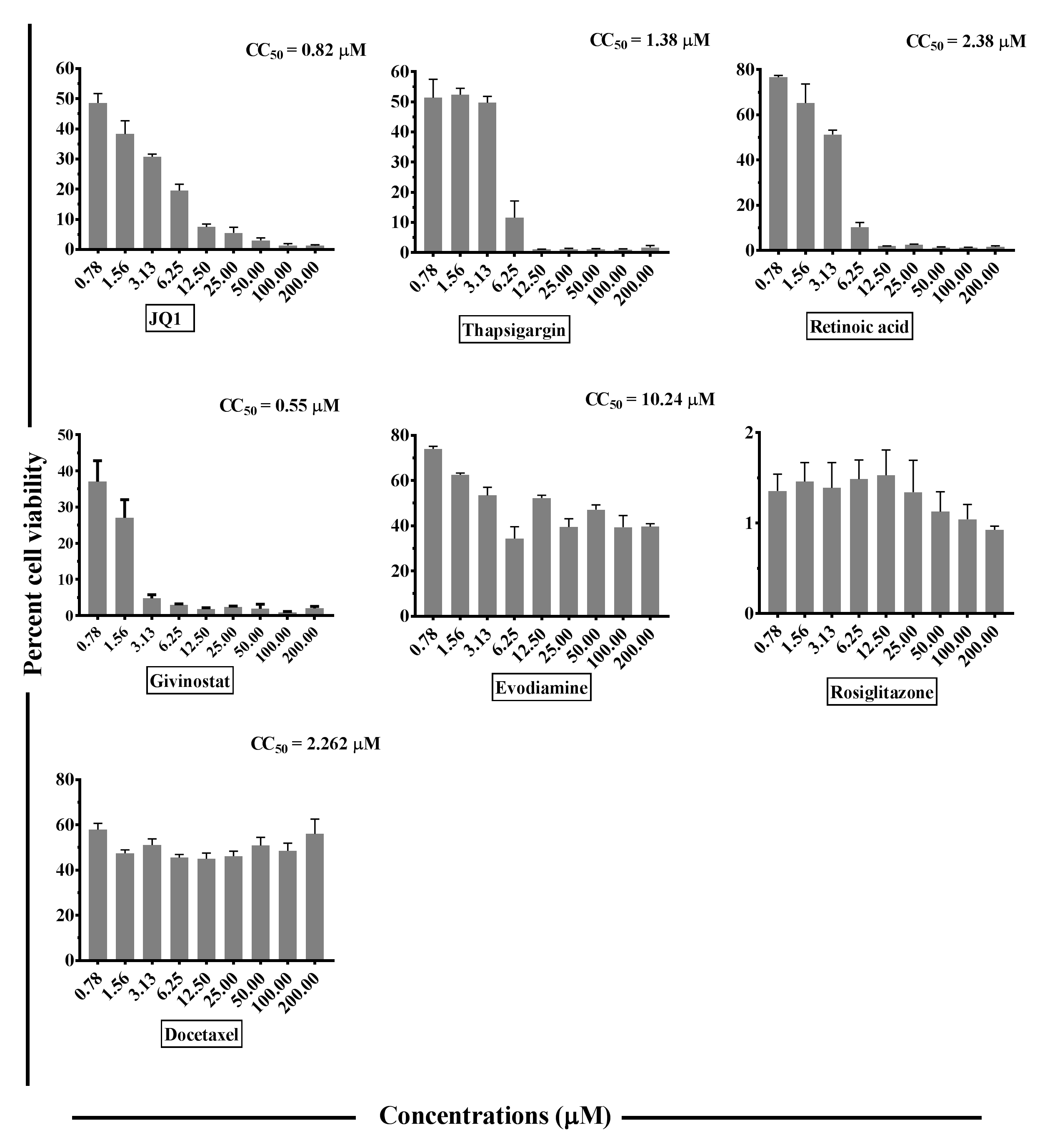

Supplement: Supplementary file 1 [file viruses-14-02150-s001.zip › Figure S1_2.tif]
